# Progress in Ophthalmology

**Published:** 1895-07-20

**Authors:** 


### Progress in Ophthalmology.

**Skin Grafting for Symblepharon.** — Dr. Talbot Chambers[1] relates a case of symblepharon, following a burn from lime, in which, after the failure of many other methods, a cure was effected by transplanting a flap of skin from the temporal region to the eyeball. A pedicle was left, and divided after four days. The patient was rendered maniacal by cocaine, which had to be abandoned.

**Pyoctanin in Ciliary Blepharitis.**—Dr. Gallmaerts[2] speaks highly of the use of pyoctanin, either as a stick or in a one or two per cent. solution, in the cure of severe cases of ciliary blepharitis. After careful cleansing of the lid edges, the stick is applied directly, or the surface is swabbed with the solution. He asserts that two or three applications, at intervals of a day or two, will cure the worst cases, and that the only disadvantage is the temporary blue discolouration of the lids.

**Tincture of Iodine in Corneal Ulcers.**—Dr. Carl Koller[3] recommends the application of tincture of iodine to obstinate or spreading corneal ulcers, and has found it efficacious when the actual cautery has produced only temporary benefit.

**The Care of Artificial Eyes.**—Dr. Trousseau[4] writes on the care which should be taken in the use of artificial eyes, in order to prevent them from becoming sources of irritation. He advises the removal of the eye every night, and that it should be placed in a tepid solution of boric acid until morning, a cold solution being apt to render the eye brittle. In the morning the cavity should be washed out with boric acid solution, the eye carefully wiped with sterile cotton wool, and the hands cleansed before introducing it. If the cavity should become irritated or painful, the eye must be laid aside for a time, and the condition treated by astringents or antiseptics.

**Conjunctival Occlusion in Wounds of the Cornea.**—Dr. de Wecker[5] recommends conjunctival occlusion in extensive wounds of the cornea. He dissects up the conjunctiva and sub-conjunctival tissue all round, as far back as to the insertions of the recti muscles, and then draws forward the detached conjunctiva as a sort of cap or cover, uniting its margins with a single purse-string suture or by vertical sutures. He then applies a bandage, which is suffered to remain until the stitches come out, usually in about eight days. The conjuctiva will peel off the uninjured portions of the cornea, leaving a bright surface, and will adhere to the wound and form an element in the cicatrix. Any deeper injury resulting from the wound, such as traumatic cataract, can be attended to after healing is complete.

**Penetrating Wounds of Ciliary Region and Lens.**—Dr. Randolph[6] advocates the performance of iridectomy and the prompt removal of the traumatic cataract in these cases, as a means by which enucleation may often be rendered unnecessary. He states the following conclusions: 1. In penetrating wounds of the ciliary region and lens, even when light perception is gone, and where usually enucleation is performed, the removal of the lens will often be followed by the recovery of comparatively useful vision. 2. The time to perform the extraction is in the first week of the injury, when there is less reason for entertaining the fear of sympathetic ophthalmia, or when sympathetic disease is too remote a contingency in any event, and certainly at this stage, to outweigh every other consideration. 3. The effect of the operation is to remove what is really a foreign body, and at the same time it frees the ciliary region of its infectious contents—very much the effect of opening an abscess. 4. Cleanliness is imperative in this operation. I usually sterilise my instruments in a two per cent. solution of bicarbonate of sodium, and keep the field of operation constantly irrigated with a two per cent. solution of boric acid. Any solutions that irritate—such, for instance, as sublimate solutions—are to be avoided, as they weaken the resisting powers of the eye. The after treatment consists in the instillation of atropine, one per cent., every four hours, and the wearing of a compress bandage. 5. Improvement in these cases, as would be expected, is rapid, and unless it is rapid one should not delay enucleation.

**Vascularised Cornea.**—Mr. Scott,[7] of Cairo, makes a suggestion that, when the vessels are few in number, the best treatment is to take a fine Graefe's knife, and with it slit up every individual vessel. He says that, in suitable cases, this gives far better results than either peritomy or syndectomy, as far as clearing the cornea is concerned.

**Evacuation of the Eyeball in Panophthalmitis.**—Dr. Truc,[8] of Montpellier, advocates the employment of evacuation in the treatment of general suppuration of the eyeball, whether traumatic or arising from other causes. He divides the cornea in the horizontal meridian, removes the two flaps with scissors, removes central purulent masses from the cavity with a curette, which is not permitted to touch the walls, and completes the evacuation by syringing with a solution of mercuric perchloride or of boric acid. The cavity is then sprinkled with iodoform, and filled with a light plug of cotton wool, steeped in the antiseptic solution and retained by a lightly-applied gauze bandage Healing is said to occur speedily, leaving an excellent stump for an artificial eye.

**A Source of Infection of the Eye.**—Dr. Thompson,[9] of Kansas, calls attention to the possibility that wounds of the eye, either surgical or accidental, may become affected by organisms proceeding from a dirty and neglected mouth, and gives illustrative cases, upon which he founds the recommendation to ascertain the state of the mouth, and, if necessary, to have it cleansed and disinfected before performing any operation upon the eye.

**Optic Neuritis in Ozæna.**—Dr. Sulzer[10] records two cases of optic neuritis, with serious diminution of vision, which occurred in patients who were the subjects of ozæna, and which could be traced to no other cause. Under treatment directed to the condition of the nose, the vision was restored, and the optic nerves returned to their normal aspect.

**Phenate of Mercury in Corneal Affections.** — Dr. Galezowski[11] recommends phenate of mercury as a substitute for the yellow oxide in affections of the cornea, and chiefly in herpetic and phlyctenular keratitis. As a lotion, he uses from 0·010 to 0·047

milligrammes of the salt to 100 grammes of distilled water; and, as an ointment, from 5 to 10 centigrammes of the salt to 10 grammes of lanoline. He disapproves of the use of cocaine in these cases, and does not employ the phenate of mercury until the congestion is diminishing under a sedative treatment. In a discussion which followed the reading of Galezowski's paper, Dr. Gorecki called attention to the variations in the action of the yellow oxide as prepared by different methods.

**Formol in Purulent Ophthalmia.**—Dr. Fromaget,[12] of Bordeaux, strongly recommends the use of formol in the purulent ophthalmia of infants. He uses a solution of 1 in 2,000 for cleansing, and of 1 in 200 for instillation. He considers formol to be equal to nitrate of silver in efficacy, and superior to it in safety and convenience. He gives thirteen cases, and refers to the paper by Valude on the same subject in the *Annales* for July, 1893.

**Ocular Headaches.**—Dr. Landman[13] describes the characters and peculiarities of the forms of headache which depend upon uncorrected errors of refraction. Migraine is rarely caused by such errors, even when it accompanies them, and the headaches caused by ocular defects are usually continuous. They are more frequent in women than in men, and can generally be localised, a diffused, continuous headache being usually due to some other cause. If the patient complains of headache about the eyes, or of a pain extending from the eyes to the occiput, or around the eyes after work with them, or at the "back of the eyes," or of throbbing, increasing towards evening, or of any of these diminished by rest, it is probable that the eyes are in fault. The author regards the headache as due to errors of refraction, and not to want of equilibrium of the motor muscles. Two hundred cases of ocular headache presented the following localisation: over the brows, 41 per cent.; on the vertex, 20 per cent.; occipital, 12 per cent., occipito-frontal, 8 per cent.; temporal, 8 per cent. In only one case was the pain general. The forehead and the vertex, the occiput and the vertex, were each the seat of pain in thirteen cases, and the occipital headaches were often attended by stiffness of the neck.

**Scopolamine as a Mydriatic.**—Dr. Walter[14] relates a case in which the use of scopolomine in irido-choroiditis was followed by an outbreak of glaucoma requiring prompt iridectomy. It has been maintained, by Raehlmann and others, that scopolamine was free from this liability; but Walter's case appears to show that its use entails the same dangers which attend the use of other similar agents.

**Salicylate of Soda in Exophthalmic Goitre.** — Dr. Chibret[15] relates four cases of Graves' disease, in which great benefit was derived from salicylate of soda, in doses averaging five grammes daily. Improvement speedily followed, but it was found necessary to continue the treatment for some time in order to avoid recurrence, but in doses gradually reduced to two grammes daily. He recommends the daily quantity to be given in four doses, and in at least half a litre of water.

**Methyl Blue in Epithelioma of the Eyelids.**—Dr. Fage[16] reports three cases of epithelioma of the lids successfully treated by the application of a solution of methyl blue, of one in twenty. In large tumours, he advises the excision of the bulk of the growth, or its destruction by the actual cautery, as a preliminary to the application.

**Suture of the Cornea in Cataract Extraction.**—The use of a suture to close the wound made in extracting cataract was originally proposed by Dr. Williams, of Boston, many years ago, and the proposal has recently been revived by Dr. Kalt and others. Kalt inserts a suture prior to operation, passing it first through the outer layer of the cornea near its summit, then through the outer layer of the sclera just beyond the corneal margin, draws the intermediate portion of thread into a loop towards the nasal side, and makes his section so as to fall between the upper and the lower portions of the thread, which, when the operation is completed, are tightened and tied. Dr. Trousseau[17] relates a case in which, on the third day after an extraction, he found the corneal flap completely everted and turned down. When replaced it returned to this position, until he inserted a suture and so secured it. The patient made a good recovery, and some vision was retained.

1 Med. Record, April 20, 1895. 2 The Med. Week, April 12, 1895. 3 New York Med. Jour., March, 1895. 4 Presse Médicale, March 2, 1895. 5 Med. Week, Feb. 22, 1895. 6 New York Med. Jour., Feb. 23, 1895. 7 Glasgow Med. Jour., Jan., 1895. 8 Med. Week, Nov. 2, 1894. 9 New York Med. Jour., Oct. 13, 1894. 10 Ann. d'Oculistique, Jan., 1895. 11 Ann. d'Ocul., Jan., 1895. 12 Ann. Ocul., Feb., 1895. 13 American Journal of Ophthalmology, Vol. XI. 14 Klinische Monatsblatter für Augenheilkunde, Jan., 1895. 15 Revue générale d'Ophthalmologie, Jan., 1895 16 La Clinique Ophth., Jan., 1895. 17 Annales d'Ocul., March, 1895.

